# Design and implementation of college students’ physical education teaching information management system by data mining technology

**DOI:** 10.1016/j.heliyon.2024.e36393

**Published:** 2024-08-15

**Authors:** Wei Rao

**Affiliations:** School of Physical Education, Wuhan University of Science and Technology, Wuhan, China

**Keywords:** Data mining technology, College physical education, Physical education teaching, Information management system, System design

## Abstract

This study intends to improve the efficiency of physical education teaching management, accelerate the normal teaching process, and meet the modern management requirements that traditional teaching management methods cannot meet. Based on data mining technology, this study designs a college student physical education teaching information management system, and makes a detailed design of each functional module. The main task of this study is to investigate how to effectively integrate data mining techniques with existing university student physical education teaching databases. Then, this study finds useful data information from massive data information to provide information support for university student physical education teaching. In order to effectively mine the relevant information of the data, the student evaluation module in the system is designed based on decision trees, and the teacher-student related data analysis module in the system is designed based on association rules. The research results indicate that 1039 records and 8205 student records are extracted from the teaching management database as mining objects. Rule 1: The support rate for “a professor's degree is a doctoral degree” is 20.4 %, indicating that there are 20.4 % of records in the teacher database that “the title is a professor and a doctoral degree”; the confidence level of Rule 1 is 78.2 %, indicating that 78.2 % of professors have a doctoral degree. Through the analysis of the rules that evaluate teaching as good, it can be found that the three attributes of professional title, education level, and teaching experience are the most important relevant factors affecting teaching effectiveness. Research has shown that the longer and richer the teaching experience, the stronger the teaching ability. Secondly, the mining results obtained through data mining techniques are analyzed. The maximum difference between the original algorithm's support mining results and the true values is 0.08, while the maximum difference between the improved algorithm's support mining results and the true values is 0.01. Compared to the original algorithm, the improved algorithm's mining results are accurate and effective. The application of data mining ideas in this system has laid a solid foundation for the development of physical education and teaching. Moreover, a three-layer system architecture model is adopted to better adapt to the development of school physical education, which is beneficial for later system maintenance and greatly reduces the work pressure of teachers. The system has been successfully launched and running in universities, and it is in good working condition.

## 1 Introduction

With the advancement and integration of computer network technology alongside the widespread availability of modern high-tech educational infrastructure, diverse network information management software has seen extensive adoption in higher education institutions. However, colleges and universities often face shortcomings in physical education (PE). Addressing these challenges requires the establishment of a corresponding data informatization system, leveraging comprehensive data mining technology for fully automated processing and implementing an expansive sports data information analysis system. Such systems streamline result verification for students and provide educators with enhanced data analysis capabilities [1]. The efficacy of this system's infrastructure extends beyond efficiently, accurately, and reliably verifying numerous results. It also encompasses the ability to classify, summarize rankings, and undertake screening tasks. Of paramount importance is its capability to develop tailored programs based on students' individual physical attributes. These programs can continually monitor students' health statuses, facilitating real-time assessments that empower students to comprehend their physical well-being, cultivate interest in sports, and infuse cultural and PE activities with substantive significance [2].

The essence of data mining lies in extracting profound insights and rules from voluminous, random, fuzzy, and incomplete datasets [3]. This technology is widely applicable across various domains, enabling the derivation of classification models, clustering models, association models, and more from diverse data sources such as relational databases, data warehouses, and textual repositories [4]. Currently, data mining has garnered significant success in fields including finance, telecommunications, bioengineering, and information retrieval [5]. Previous studies on college teaching management systems have often relied on statistical analysis methods. While effective for analyzing historical data to identify teaching patterns and trends, these methods may have limitations in predicting future changes, handling nonlinear relationships, and optimizing complex problems. In contrast, the integration of machine learning and metaheuristic hybrid methods offers a promising approach. By learning from historical data and seeking optimal solutions through simulation and optimization, these methods can potentially uncover hidden correlations and adjust teaching strategies in real-time to adapt to evolving environments and demands. In the design and implementation of a college PE teaching information management system, leveraging machine learning and metaheuristic hybrid algorithms presents several advantages. Firstly, machine learning can tailor exercise recommendations and teaching content based on extensive student physical fitness, performance, and learning behavior data, thereby enhancing teaching precision [6]. Secondly, metaheuristic algorithms can tackle complex optimization problems like course scheduling and facility allocation. When combined with machine learning predictions, they can dynamically adapt and proactively adjust teaching strategies. Moreover, the hybrid algorithm can enhance system performance in handling large-scale complex problems by integrating real-time learning from machine learning and global optimization from metaheuristics. This foresighted decision support enables teaching managers to efficiently respond to various changes, thereby comprehensively improving the operational efficiency and teaching effectiveness of PE teaching information management systems [7].

In recent years, hybrid algorithms combining machine learning and metaheuristics have demonstrated significant potential, particularly in educational management. This study examines relevant research, analyzing methodologies, application domains, and outcomes to offer insights for future studies. Yağcı (2022) introduced an intelligent education management system leveraging machine learning and data mining. Their approach involved using machine learning algorithms to analyze and forecast students' learning behaviors, while employing data mining techniques to reveal hidden patterns in learning data. Experimental validation confirmed the system's effectiveness in educational management, furnishing a scientific foundation for educational decision-making [8]. Dasi (2024) investigated methods to improve the performance of metaheuristic algorithms in dynamic optimization problems using machine learning techniques. He proposed a hybrid approach combining metaheuristic algorithms (e.g., genetic algorithms, particle swarm optimization) with reinforcement learning or deep learning. This integration enables algorithms to adapt to environmental changes in real-time, enhancing optimization effectiveness in uncertain and dynamic settings [9]. Abdollahzadeh (2024) concentrated on merging the predictive and learning capabilities of neural networks with the global search ability of evolutionary algorithms, a typical metaheuristic. Their novel hybrid algorithm leverages neural networks to learn from historical search experiences, guiding the optimization process, while evolutionary algorithms handle search operations. This synergy accelerates convergence to optimal solutions in complex tasks and improves generalization ability for new problems [10]. The integration of insights from these studies underscores the widespread application and considerable potential of machine learning and metaheuristic hybrid algorithms in educational management. These findings offer crucial references and insights for future research in related fields, fostering innovation in PE teaching information management as discussed in this study.

This study aims to address prevalent challenges in college PE teaching management, specifically the uneven distribution of teaching resources and the non-standardized management of students' physical data. Disparities in resource allocation can affect teaching quality, while non-standardized data management may hinder a comprehensive understanding of students’ physical conditions. The central inquiry of this study is how to leverage data mining technology to develop an intelligent management system. This system aims to optimize resource allocation and standardize student physical data management, thereby improving the efficiency and quality of college PE teaching management [11].

Drawing upon data mining technology as its cornerstone, this study initiates the design of the system architecture and database, utilizing a data mining platform for system realization in its sub-modules. Subsequently, the study undertakes an in-depth analysis and discussion of the data results generated by the newly developed system. In the contemporary academic landscape, numerous institutions of higher learning are exploring system software, integrating data mining technology into teaching and education management systems, thereby enhancing students' physical fitness and raising the standard of school teaching management. Responding to challenges faced by colleges and universities, a proposed framework for a management system assessing students' sports performance has been introduced [12]. The primary objective is to use the system to address prevailing issues in PE within academic institutions, fostering enthusiasm for physical exercise among students, developing robust physical capabilities, and ensuring the overall quality and efficacy of school teaching. The study comprises four main sections: the introduction, methodology, results and discussion, and conclusion. The introduction provides the study background and objectives, summarizes the current state of research and technology through a literature review, and outlines the study's contributions [13]. The methodology section introduces the technical methods and background, including the design of the system architecture and database, the role of data mining, module design, and the establishment of the implementation platform and experimental environment. The results and discussion section summarizes experimental outcomes, including unit tests, stress tests, and program debugging analyses. The conclusion section summarizes the main research findings and contributions and indicates prospects for future research.

This study aims to rectify the disparities in teaching resource allocation and the lack of standardized management of student physical data in current PE teaching management. It seeks to accomplish this by developing a college PE teaching information management system based on data mining technology. Through this system, intelligent allocation of teaching resources and efficient management of student physical data can be achieved, ultimately enhancing the effectiveness of teaching management. The study's contribution lies in proposing an innovative solution and designing and implementing a corresponding prototype management system, thereby offering fresh perspectives and methodologies for college PE teaching management.

## 2 Method

### 2.1 System architecture and database design

**Fig. 1:**
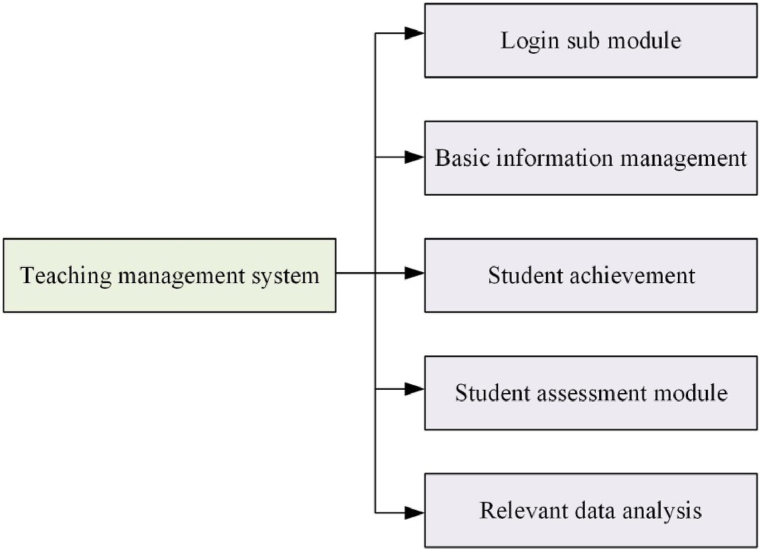
Decision-making function of college students' PE management.

The software engages in the analysis, processing, and mining of data pertaining to college students, serving as a tool for PE managers. This study advocates for the incorporation of essential components within the PE management system for college students. These components encompass a login mechanism [14], basic information management [15], student performance management [16], student evaluation management [17], and a data management system concerning teacher-student relationships [18]. The foundational framework of the college PE teaching and management decision-making system is delineated, as illustrated in Fig. 1.

In Fig. 1, the login module is utilized for user authentication and system access. The basic information management module oversees fundamental information concerning students, teachers, and courses. The student performance management module records and analyzes students' PE achievements. The student evaluation management module collects and analyzes students’ assessments and feedback on PE teaching. The teacher-student related data management system oversees interactive data between teachers and students, such as teaching schedules and assignment submissions. These modules are interconnected, constituting a comprehensive PE teaching and management system that offers support and data analysis for higher education PE teaching and management decision-making.

**Fig. 2:**
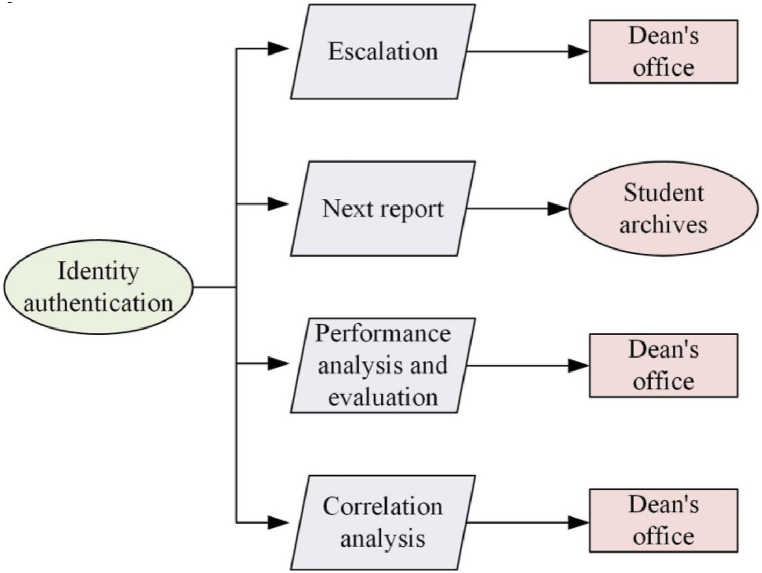
Business process of college PE teaching management decision-making system.

The business process design of the college PE teaching management system is illustrated in Fig. 2 [19]. In Fig. 2, the business process consists of multiple interrelated entities and steps, which work together to support and optimize university physical education teaching and management decisions. The specific content and its relationship are as follows: Identity Authentication: Users can only enter the system after passing identity verification, which ensures the security of the system and the confidentiality of data. Escalation: After identity authentication, users can report data and submit relevant information to the academic affairs office for review and processing. Dean's Office: The Dean's Office is responsible for receiving and processing reported data, ensuring the accuracy and timeliness of the data. Next Report: After processing the current data, the Dean's Office prepares the next data report. Student Archives: The system contains detailed student archives information, recording basic information, physical education scores, evaluation feedback, and other data of students. Performance Analysis and Evaluation: The system analyzes and evaluates students' physical education performance, generates detailed reports and trend analysis, and provides reference for teaching improvement. Based on the analysis and evaluation of grades, the Dean's Office makes corresponding teaching adjustments and improvements. Correlation Analysis: The system conducts correlation analysis on various types of data, discovers potential relationships and patterns between data, and provides deep data insights. Based on the results of correlation analysis, the Dean's Office further optimizes the allocation and management of teaching resources to improve teaching effectiveness; These steps form a closed-loop business process, continuously optimizing and improving the management of physical education teaching in universities through identity authentication, data reporting, processing and analysis, report generation, feedback adjustment, and other links.

**Fig. 3:**
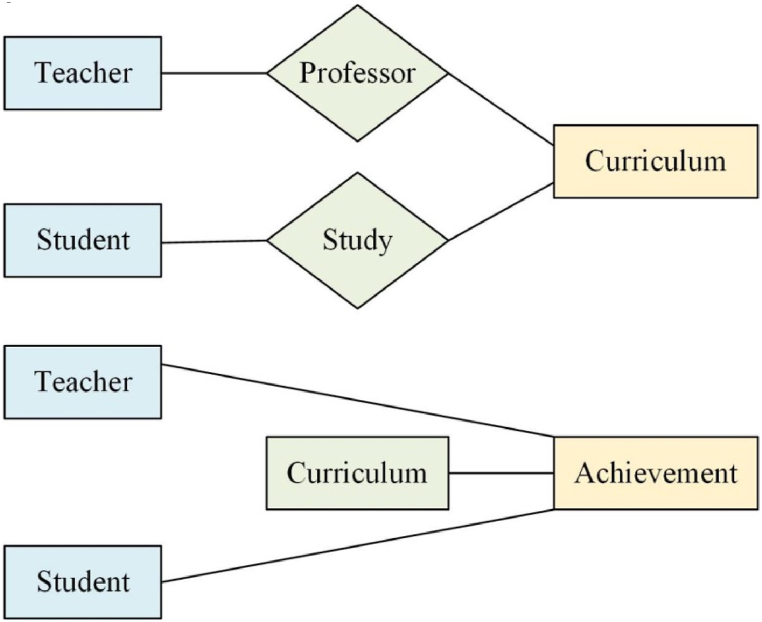
E–R diagram.

In Fig. 2, the system encompasses entities such as students, courses, and teachers. Each entity is characterized by distinct attributes, with a notable “course selection” relationship established between the “student” and “course” entities [20]. The Entity-Relationship (E-R) diagram is presented in Fig. 3.

The E-R diagram serves as a means to represent entity types, attributes, and relationships, offering a conceptual model of the real world. It proves to be an efficient method for describing conceptual models of real-world relationships, serving as a representation of conceptual relational models. The “rectangular box” signifies the entity type, with the entity name inscribed within. The “diamond box” denotes the causative factor of the connection between entity types, with the connection name specified in the diamond box. The “solid line segment” establishes a connection with the relevant entity type, with the type of connection annotated on the “solid line segment.”

### 2.2 Role of data mining in the system

The existing decision-making system is currently built upon the framework of the management information system. Integrating this technology into the decision support system for teaching management in higher education institutions poses several challenges:

(1) Data disparity: Operational data managed by teaching management decision-making systems in higher education institutions are typically diverse and heterogeneous. The effectiveness of the decision support system depends on synthesizing and dynamically integrating these dispersed data types [21].
(2) Lack of unified standard: The data processed by the teaching management decision-making system lacks a unified standard, hindering its conversion into actionable information for decision-making purposes [22].
(3) Limitations in handling large-scale data: Conventional decision support systems often struggle with large-scale data, offering only basic small-scale data aggregation and processing. Consequently, these systems may provide inadequate or redundant information for decision-making, failing to meet the comprehensive needs of a decision support system [23].

The college PE teaching management decision-making systems faces limitations in addressing common qualitative, fuzzy, and uncertain decision-making issues [24].

Data mining technology, capable of extracting valuable knowledge and rules from extensive datasets [25], utilizes artificial intelligence, statistical methods, or other algorithms to uncover strategic insights [26]. Integrating data mining techniques into decision support systems for teaching management in higher education institutions is crucial for three main reasons [27]:

(1) Extraction of valuable information: Applying data mining technology allows managers to extract valuable insights from accumulated data, offering commercially relevant insights. This process involves identifying pertinent features or patterns during data mining for teaching management decisions [28].
(2) Enhanced information provision: Integrating data mining technology into the teaching management decision support system enhances the delivery of more effective information, thereby increasing the level of management automation and alleviating the managerial burden [29].
(3) Utilization of data warehousing: Applying data mining technology in the decision support system for teaching management leverages the capabilities of a data warehouse. This repository can store substantial volumes of data, comprehending the outcomes and reasons for the success or failure of all recorded executions in the past and present. Its analytical processing capabilities contribute to the extraction of decision-relevant knowledge [30].

### 2.3 Module design

#### 2.3.1 Design of the login sub-module

**Fig. 4:**
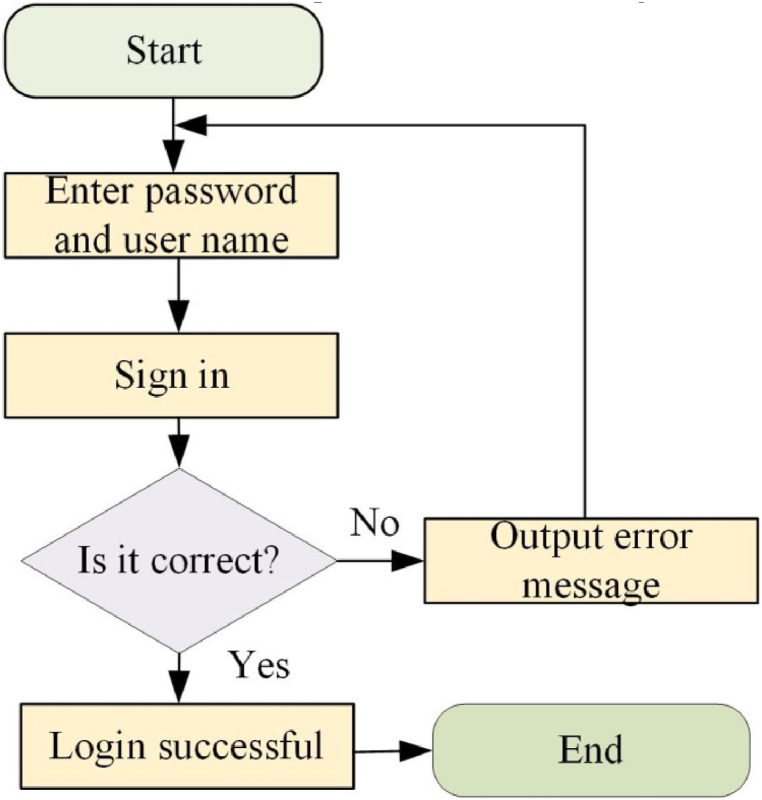
Login process.

The login module primarily allocates distinct functional menus based on the user's login credentials. System users fall into two categories: those who are not logged in and those who have successfully logged in. Logged-in users include administrators and teachers [31]. The procedure for accessing this module is illustrated in Fig. 4:

The software's procedural design revolves around the login module's functionality. Users input their account names and passwords into the module and click the “login” button. The program then verifies the entered account name and password for authentication. Upon correct input, the screen displays the message “login successful.” If incorrect user name and password entries occur, or if the initial user name and password are used, an error message is prompted.

#### 2.3.2 Development of information management sub-modules for teachers and students

**Fig. 5:**
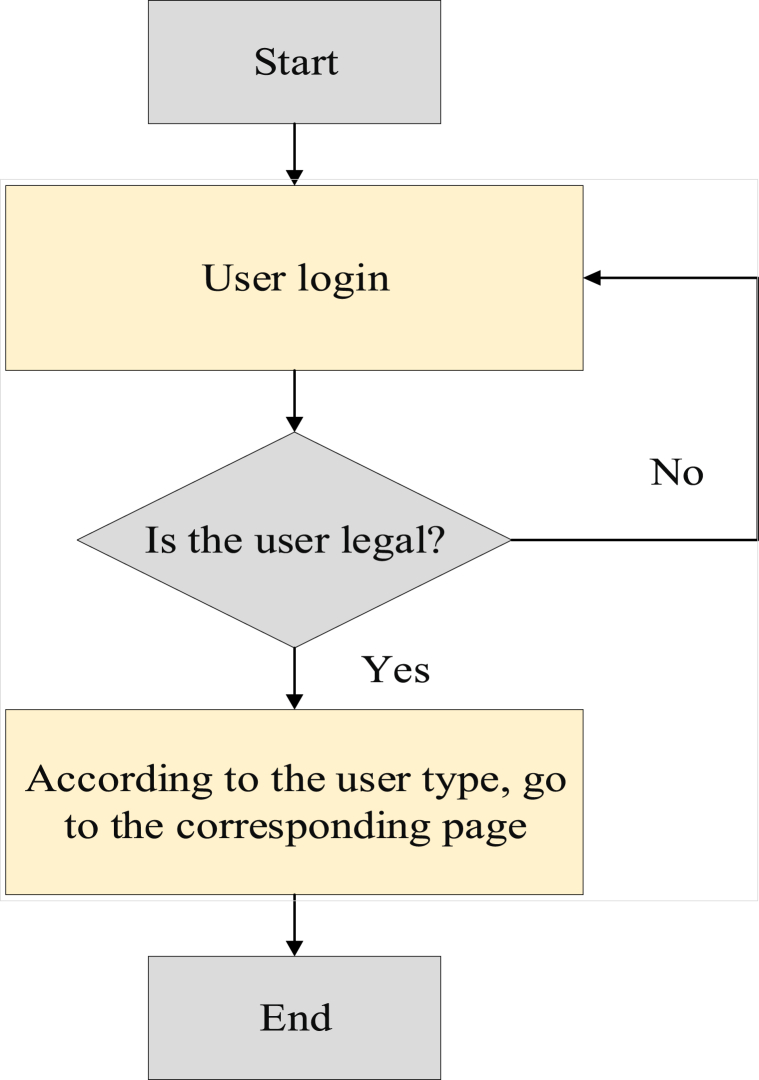
Flow of the login function.

This system enables teachers to manage student information by facilitating data entry, modification, and deletion through system operations. The user base includes administrators and teachers [32]. The procedural steps for implementing the registration interface are illustrated in Fig. 5.

This module operates sequentially, commencing with the login process. On the login page, the program verifies the entered user name and password, ensuring alignment with the provided credentials. In case of a mismatch, the system returns to the initial interface. Upon correct credentials, users gain access to the relevant webpage based on their category and authorization, subsequently reaching the judgment webpage.

#### 2.3.3 Establishment of the student achievement database management module

**Fig. 6:**
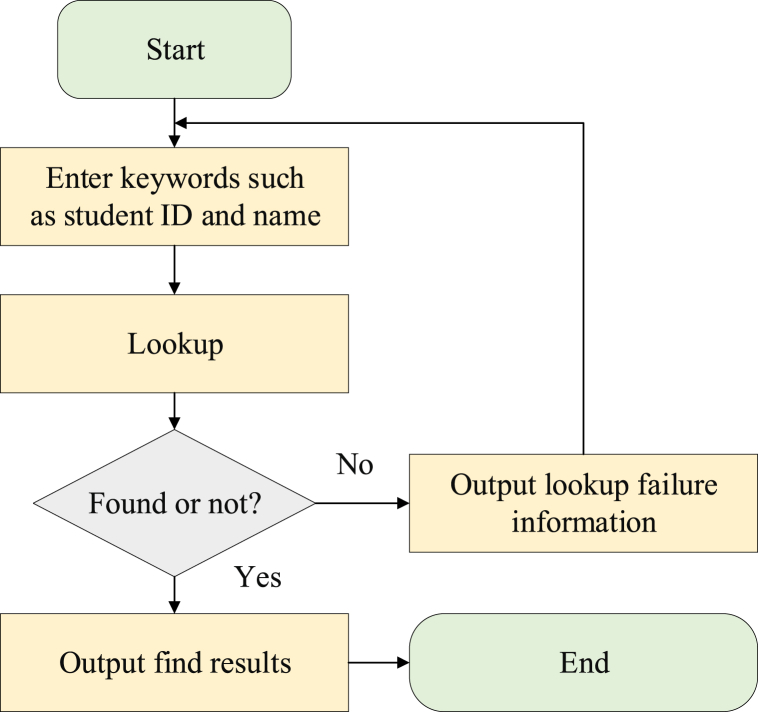
Query process of the student grade database.

This unit is designed to configure student grades, identification numbers, classes, majors, credits, grades, re-examinations, makeup examinations, and related information. The functionality of the student grade database includes data entry, modification, and query operations [33]. The precise implementation process of the student grade database module is outlined in Fig. 6.

In Fig. 6, upon initiation of the software, students input details such as their student ID and name. The database serves as a tool for querying and displaying student information.

If there is no available information for the specified student, the system prompts the user to enter the student ID and name again.

### 2.4 Realization platform of the system

**Fig. 7:**
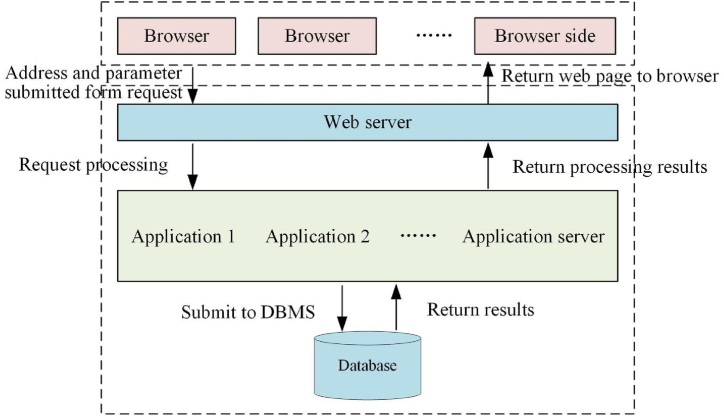
Schematic representation of the B/S model used in the system.

The system design is executed in the B/S mode, involving the establishment and maintenance of the back-end database, along with the development of the front-end application, as supported by prior studies [34,35]. The configuration and interrelation of the B/S model are illustrated in Fig. 7. The primary technology employed for system development is Active Server Pages (ASP).

In Fig. 7, the user-submitted form, which entails requests for address and parameters from the browser, undergoes solicitation and processing by the Web server. Subsequently, the application is initiated, and the database is processed accordingly. The outcome is conveyed back to the application server, and the processing result is then returned to the Web server. Ultimately, the web page is transmitted back to the browser side.

System Development Platform:

(1) The network server operating system is Windows 2000 Server.
(2) The Web server employed is Internet Information Server 5.0.
(3) The background database system utilized is Microsoft Access.
(4) The web programming languages encompass HTML, ASP, and VBScript.
(5) Front-end editing tools for web pages include EditPlus2 and Microsoft Office Front Page2000.

Implementation of the Login Submodule:

The user base for this system is categorized into two groups: those who have not logged in and those who have successfully logged in. Logged-in users comprise administrators and teachers. The delineated function menu is presented in Table 1.

**Table 1:** Function menu.

Users	Teacher	Administrator
Function Menu	1. View course offerings 2. View Student Assessment Information 3. View relevance assessment information 4. Add course grades 5. Check the class grades 6. Check course grades	7. View Student Assessment Information 8. View Relevance Assessment Information

### 2.5 Experimental environment

The choice of experimental environment has a significant impact on the effectiveness of system development and implementation. Table 2 shows the server configurations required for the development of this system.

**Table 2:** Configuration of the server.

Configuration Item	Number	Performance	Demand Stage
Server	1	Standard CPU quantity: 1; 8 GB memory; CPU model: XeonE5506; dual-port 10 Gigabit network card; supports up to 2 SFFSATA/SAS/SSD hard drives; RAID5	Code Writing Stage
Client Computer	50	CPU model: Intel Pentium dual-core G630; 2 GB memory; hard disk capacity: 500G; 1000 Mbps Ethernet card	Code Writing Stage

Server configuration:

The configuration of the server mainly considers the operational efficiency and stability of the system. Choosing a server with standard CPU and 8 GB of memory can provide sufficient computing and storage capabilities during system development and testing phases. The choice of XeonE5506 CPU is based on its efficient performance in handling multi-threaded tasks. The dual port 10-Gigabit network card ensures the speed and stability of data transmission, while the support for RAID 5 configuration provides data redundancy and protection, ensuring the security and reliability of system data.

Table 3 shows the software configurations required for the development of this system.

**Table 3:** Configuration of server software.

Configuration Item	Number	Performance	Demand Stage
Server	1	Standard CPU quantity: 1; 8 GB memory; CPU model: XeonE5506; dual-port 10 Gigabit network card; supports up to 2 SFFSATA/SAS/SSD hard drives; RAID5	Code Writing Stage
Client Computer	50	CPU model: Intel Pentium dual-core G630; 2 GB memory; hard disk capacity: 500G; 1000 Mbps Ethernet card	Code Writing Stage

In terms of server software configuration, Microsoft Access is chosen as the backend database system mainly because of its ease of use and good compatibility, which is suitable for the data storage and management needs of small and medium-sized systems. The installation of the OFFICE suite and antivirus software ensures document processing and system security during the development process. Choosing IE6.0 as the browser, although the version is older, can provide sufficient compatibility and stability during system development and testing.

The configuration of the college student physical education teaching information management system designed in the experimental environment not only considers the coordination and cooperation of hardware and software, but also pays attention to the actual needs in the system development process, ensuring the efficiency and stability of the system in the development, testing, and operation stages.

## 3 Results and discussion

### 3.1 Analysis of unit test results

In the system testing context, unit testing emerges as the most straightforward phase, applicable to any stage of the system. “Unit testing” involves isolating each functional module into distinct units and testing them independently to ensure they achieve their intended objectives, thereby identifying potential issues and verify the correctness of the program in real-time performance.

After the programmer completes the program, corresponding tests are conducted to ensure its seamless operation. The programmed test cases serve to further verify the correctness of the program.

Throughout the development of the target system, diverse functional and programming units undergo comprehensive testing. These selected units are thoroughly explored, incorporating various data states, both abnormal and normal, to enhance the precision of the testing process.

During testing, examination of relevant functional modules or system programs typically reveals issues. Rectifying these identified problems at this stage is relatively cost-effective. However, if these issues remain undetected, it not only escalates costs but also increases the workload. In the unit testing process, the target system encountered certain issues.

Each module undergoes meticulous testing. Due to space constraints, this study selectively analyzes some typical cases and provides representative instances.

Table 4 outlines the test case employed during the unit testing of the system login function module.

**Table 4:** Description of system test cases.

Instructions	Test Data	Expected Outcome
Enter incorrect password and username into the system, and attempt to log in.	Incorrect password and username	The system fails to log in and prompts error type.
Enter correct password but wrong username during testing.	1. Correct password and wrong username. 2. Correct password and empty username. 3. Correct password and username with special symbols. 4. Correct password and incorrect user.	1. The system cannot log in and prompts to re-enter the correct password or username. 2. The system prompts us to re-enter the username. 3. The system cannot log in and prompts to enter the correct password or username. 4. The system cannot log in and prompts to enter the wrong password or username.
Ditto	Ditto	Ditto
Enter correct password and username for system login.	Correct password and username.	The system successfully logs in.

The test of the college students' PE information management module is presented in Table 5.

**Table 5:** Test of college students’ PE teaching information management module.

Number	1	2	3	4
Test Content	Input has malformed data.	Empty input has required fields.	End the new operation.	Click on the “Grade Management” menu.
Actual Results	Normal trigger format validation event.	Normal trigger empty judgment event.	Display the newly added records in the first line, and display all records normally.	Show all records normally.
Expected Outcome	Unable to save, prompts for correct format.	Unable to save, prompts null value.	Add a grade record and jump to the grade management page.	Read all grade records in the database.

In practical applications, unit testing plays a pivotal role as it can effectively uncover numerous potential issues within a program. During the unit testing of the PE management system, certain issues surfaced, such as:

Issue: When attempting to save scores into the system, the examiner receives an error message stating “the entered data is incorrect.” Further examination reveals that mandatory data fields were left unfilled.

Resolution: To address this issue, the validation of mandatory items can be achieved by incorporating the required field validator control from the .NET framework on the new page.

### 3.2 Analysis of the results of the stress test

In system testing, the concurrency test is a pivotal component of the comprehensive stress examination. It involves progressively increasing the volume of accesses within a specified timeframe until a system crash occurs, serving as the termination condition. This method helps identify the system's maximum access threshold and any deficiencies. During stress testing, meticulous consideration of key issues is imperative, including:

1. Test environment: The test environment encompasses network parameters, software, and hardware configurations. Elements such as network bandwidth, physical location during testing, computer peripherals (e.g., screens and keyboards), database components, and operating system software collectively contribute to the network, hardware, and software test environments. Test results may vary depending on the testing environment. Therefore, incorporating diverse environments into system testing is crucial to yield accurate results and enhance system robustness.
2. Test data: Test data refers to the information input into the computer system to assess its performance under specific conditions. This selection includes both normal and abnormal data. Given the unpredictable nature of user-input data, a variety of potential data types must be considered. The system's ability to rapidly identify and process user-entered data signifies normal operation. Additionally, the selection of test data should encompass both random and typical scenarios to facilitate comprehensive system detection.

In the system's stress test, the establishment of initial data precedes the actual test. The system randomly selects pre-saved initial data during testing, aligning with the designer's predetermined evaluation baseline. This baseline facilitates the analysis and interpretation of test results in accordance with the designer's specifications.

3. Test steps: Test steps outline the sequence of actions undertaken throughout the testing process. These steps involve establishing test objectives, determining the work direction, selecting test tools and methodologies, formulating and executing test cases, and documenting various data. Developers provide comprehensive documentation of test results, enabling system enhancements based on derived insights.

### 3.3 Result analysis of program debugging

**Fig. 8:**
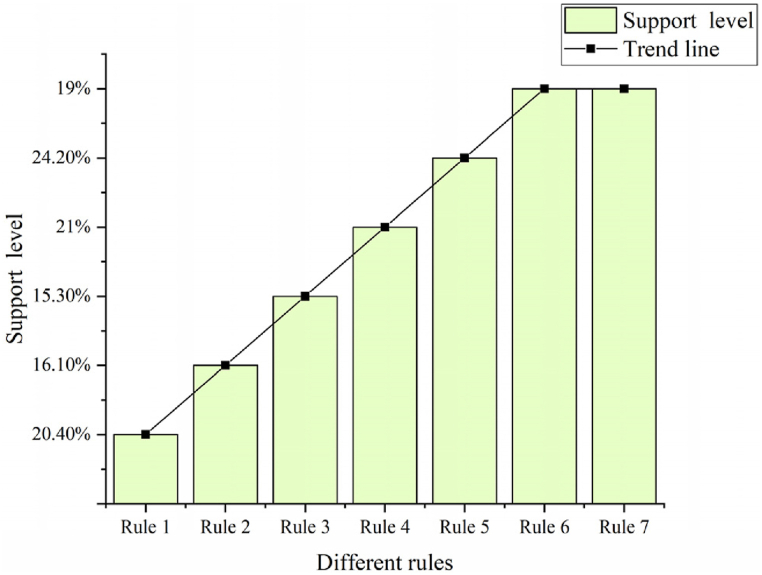
Support of different rules.

**Fig. 9:**
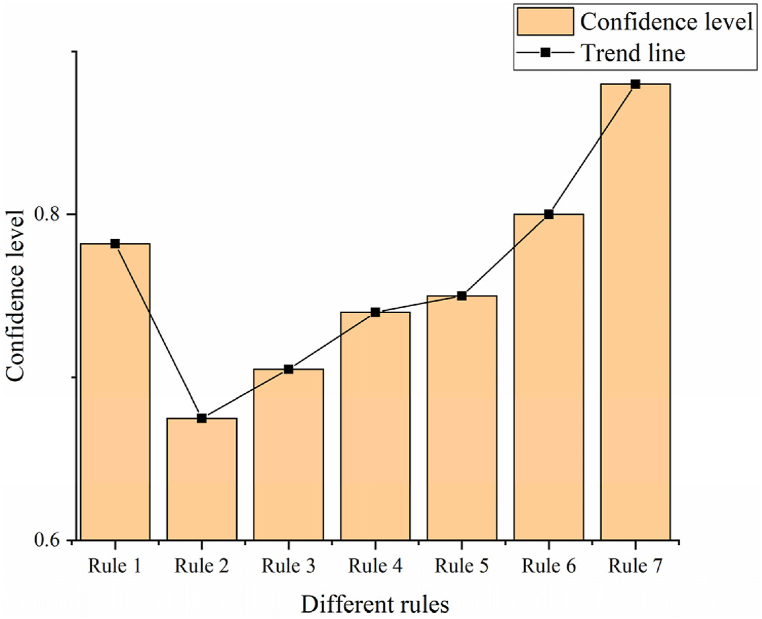
Confidence in different rules.

The program debugging is executed within the devised system. A total of 1,039 student records are extracted from the teaching management database to serve as mining objects. The corresponding rules, support, and confidence metrics are presented in Fig. 8, Fig. 9.

Rule 1: Professors with a Ph.D. in education.

Rule 2: Lecturers with over 15 years of teaching experience and an excellent evaluation.

Rule 3: Associate professors rated as excellent, with teaching experience ranging from 6 to 10 years.

Rule 4: Associate professors with 6–10 years of teaching experience are rated as good.

Rule 5: Associate professors with teaching experience between 11 and 15 years and holding a master's degree.

Rule 6: Associate professors with a master's degree and 11–15 years of teaching experience.

Rule 7: Lecturers with a master's degree and a good evaluation.

**Fig. 10:**
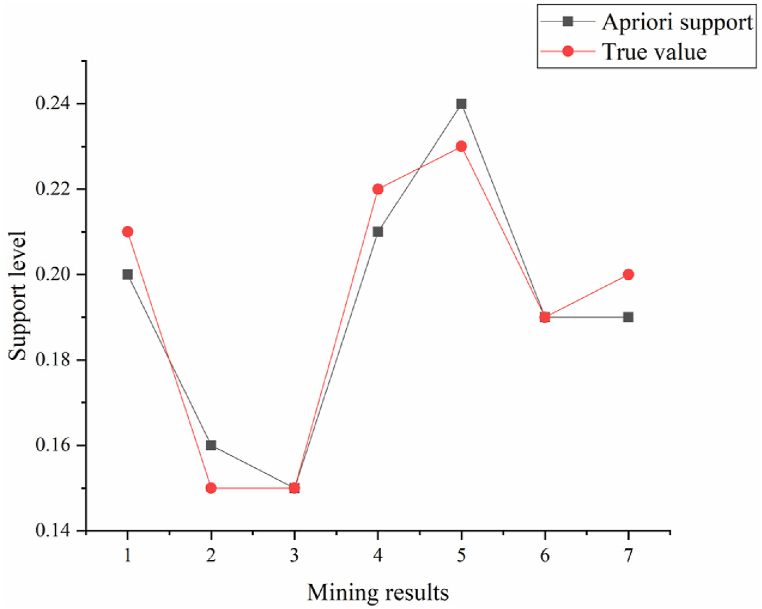
Comparison of improved algorithm support and true value.

**Fig. 11:**
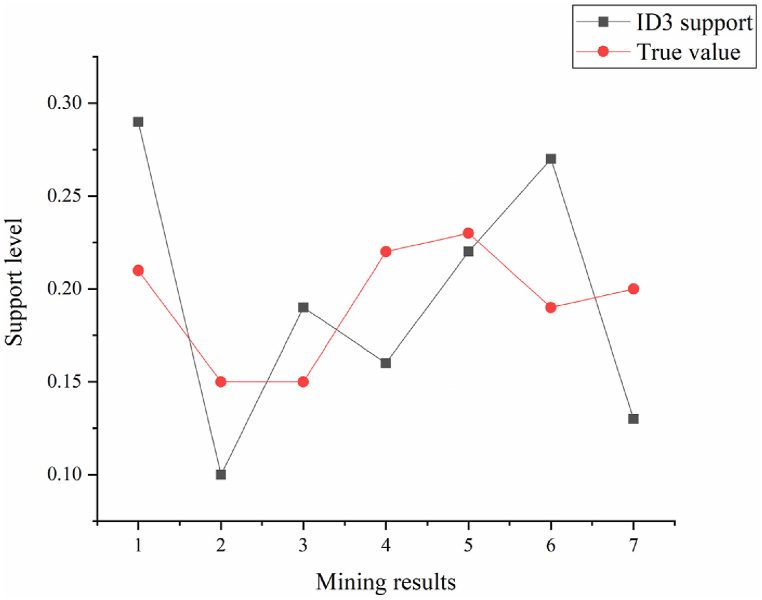
Comparison of original algorithm support and true value.

In Fig. 8, Fig. 9, Rule 1 demonstrates a support degree of 20.4 %, signifying that 20.4 % of the teacher database comprises individuals with the attribute “professional title and education degree as Ph.D.” The confidence degree stands at 78.2 %, indicating that 78.2 % of professors possess a Ph.D. An analysis of rules concerning good teaching evaluations highlights the pivotal role of professional title, educational background, and teaching experience in teaching effectiveness. Rule 4 suggests that lecturers with 6–10 years of teaching experience and the lecturer title exhibit good teaching effectiveness. Rules 2, 3, and 4 underscore a direct correlation between teaching effectiveness and the duration of teaching experience. This correlation stems from the enrichment of teaching expertise and enhancement of teaching abilities associated with longer teaching experience. The outcomes derived from data mining technology undergo analysis, comparing the original algorithm with the improved algorithm, as depicted in Fig. 10, Fig. 11.

Fig. 10 illustrates the comparison between the support values generated by the improved algorithm and the true values. The support values obtained from the enhanced algorithm are 0.2, 0.16, 0.15, 0.21, 0.24, 0.19, and 0.19, with variances from the actual values of 0.01, 0.01, 0.00, 0.01, 0.01, 0.00, and 0.01, respectively. The maximum observed discrepancy is 0.01.

In Fig. 11, the comparison between the support values derived from the original algorithm and the true values is presented. The support mining results from the original algorithm are 0.29, 0.10, 0.19, 0.16, 0.22, 0.27, and 0.13. Deviations from the actual values are 0.08, 0.05, 0.04, 0.06, 0.01, 0.08, and 0.07, respectively, with the maximum discrepancy being 0.08.

The Apriori algorithm is used to generate frequent itemsets, and its frequency is determined based on the support of the itemsets. Subsequently, decision trees are used to mine frequent itemsets and reduce the number of data scans, thereby improving mining efficiency. In specific applications, the Apriori algorithm is first used for preliminary screening to generate frequent itemsets. Then, decision trees are used to further mine and analyze these frequent itemsets. The application of this hybrid algorithm effectively improves the efficiency and accuracy of the system in processing large-scale data. By comparing the support mining results of the original algorithm and the improved algorithm, it is found that the maximum difference between the support of the improved algorithm and the actual value is 0.01, while the maximum difference between the original algorithm is 0.08. This indicates that the improved algorithm outperforms the original algorithm in terms of accuracy and efficiency.

### 3.4 Discussion

Wang (2023) presented the design and implementation of a PE teaching management system based on data mining technology. Through data mining algorithms analyzing students' physical data and the allocation of teaching resources, intelligent teaching management was achieved. This study is closely related to the issues addressed in this study, providing a reference for system design and implementation [36]. Gazali (2023) introduced the construction and application of a college PE teaching information management system. Through this system, the management and allocation of students' physical data and teaching resources can be realized, enhancing the efficiency and level of PE teaching management. Their research provides practical cases and application experiences that align with the goals and methods of this study [37]. In comparison with the mentioned studies, this study focuses on addressing specific issues in PE teaching management, such as uneven distribution of teaching resources and improper management of students’ physical data, demonstrating stronger specificity and practicality. Compared to the aforementioned studies, this study is innovative, proposing a solution based on data mining technology and designing and implementing a corresponding management system prototype, showcasing practical value. Additionally, this study not only emphasizes theoretical exploration but also conducts systematic system design and implementation, along with on-site testing and evaluation, demonstrating a certain level of comprehensiveness and operability.

## 4 Conclusion

Utilizing data mining technology and a teacher-student database, this study investigates the effective integration of data mining with the existing PE database of college students. The aim is to uncover valuable data information within the extensive dataset, providing informational support for college students' PE teaching. To efficiently extract relevant data information, the decision tree serves as the foundational framework for designing the student evaluation module in the system. The key findings of this study include: (1) Rule 1, “professor's education is a doctor,” exhibits a support degree of 20.4 %, signifying that 20.4 % of records in the teacher database correspond to “professor with a doctoral degree.” Rule 1's confidence level is 78.2 %, indicating that 78.2 % of professors possess a doctorate. The analysis of teaching evaluation rules emphasizes the significance of professional title, educational background, and teaching experience in influencing teaching effectiveness. The data illustrates that prolonged teaching experience correlates with increased teaching expertise, enriching teaching abilities, and enhancing effectiveness. (2) Data mining techniques are employed for analysis. The difference between the original algorithm's support mining result and the actual value is a maximum of 0.08. In contrast, the improved algorithm's support degree exhibits a difference of at most 0.01 from the real value. The enhanced algorithm proves to be more accurate and effective compared to the original algorithm. (3) The teaching information management system, designed based on data mining technology, features standardized and comprehensive basic information settings. It facilitates flexible, convenient, and rapid information querying, ensuring system stability, safety, and reliability. This study equips college PE information managers with deeper insights and rules within the database, offering targeted data support for decision-making in college PE.

Currently, the system adequately addresses the requirements of college PE management. However, certain aspects necessitate refinement: (1) Despite encompassing functionalities such as authentication, role delineation, and authorization division, the system's security remains suboptimal. (2) The database exhibits redundancy by storing identical data across different tables, impacting its efficiency due to individual capacity constraints. (3) Further enhancements are required for the software's functionality, especially concerning manual data input during data entry. (4) Accumulated data within the teaching system, spanning a considerable timeframe, is underutilized, emphasizing the need for improvements to enhance its efficiency. In summary, the system has achieved initial completion across various dimensions, but future efforts will focus on continuous enhancements to the data, offering valuable support for PE teaching in colleges and universities.

The integration of machine learning and metaheuristic hybrid algorithms offers significant promise for enhancing optimization problem-solving at a theoretical level. This amalgamation can improve performance by facilitating more precise decision-making through machine learning assistance in metaheuristic algorithms, thereby accelerating convergence and potentially discovering higher-quality solutions. In terms of computational complexity, hybrid algorithms can utilize machine learning for intelligent initialization and effective search path guidance, thus reducing aimless search attempts and shortening overall computation time. Moreover, by learning from historical data, hybrid algorithms can better adapt to new scenarios, exhibit stronger generalization abilities, and provide more refined and efficient decision support for PE teaching management. Consequently, this fusion technology is anticipated to diminish runtime overhead, boost the efficiency of solving large-scale problems in practice, and offer more comprehensive and nuanced solutions for optimizing PE teaching information systems. However, the actual effectiveness in practical applications still relies on specific algorithm implementations and empirical research validation.

While this study contributes valuable insights, it also exhibits certain limitations. Theoretical shortcomings include a lack of comprehensive analysis and discussion on the principles and algorithms of data mining technology, limiting the depth of theoretical exploration. Moreover, the theoretical framework of PE teaching management overlooks alternative perspectives and methodologies, leading to a constrained theoretical foundation. In practical terms, limitations arise from constraints on data collection and system implementation. Data acquisition constraints hindered the acquisition of sufficiently comprehensive and representative data, impacting the reliability and generalizability of the findings. Additionally, technical or resource constraints during system implementation resulted in practical limitations, such as suboptimal performance and incomplete functionality.

## Data availability statement

Data will be made available on request.

## CRediT authorship contribution statement

**Wei Rao:** Writing – original draft, Visualization, Validation, Software, Resources, Formal analysis, Data curation, Conceptualization.

## Declaration of competing interest

The authors declare that they have no known competing financial interests or personal relationships that could have appeared to influence the work reported in this paper.
